# Butylglyceryl Pectin Nanoparticles: Synthesis, Formulation and Characterization

**DOI:** 10.3390/polym11050789

**Published:** 2019-05-02

**Authors:** Mohammad F. Bostanudin, Mosab Arafat, Muhammad Sarfraz, Dariusz C. Górecki, Eugen Barbu

**Affiliations:** 1 College of Pharmacy, Al Ain University of Science and Technology, Abu Dhabi 112612, UAE; 2 School of Pharmacy and Biomedical Sciences, University of Portsmouth, St Michael’s Building, Portsmouth PO1 2DT, UK; darek.gorecki@port.ac.uk (D.C.G.); eugen.barbu@port.ac.uk (E.B.); 3 College of Pharmacy, Al Ain University of Science and Technology, Al Ain 64141, UAE; mosab.arafat@aau.ac.ae (M.A.); muhammad.sarfraz@aau.ac.ae (M.S.)

**Keywords:** polysaccharides, alkylglycerols, *N*-butylglycidyl ether, pectin, nanoparticles

## Abstract

Pectin is a polysaccharide with very good gel forming properties that traditionally has found important applications in foods and pharmaceutical industries. Although less studied, chemical modifications of pectin leading to a decrease in its hydrophilicity can be useful for the development of novel drug carriers. To this aim, butylglyceryl pectins (P-OX4) were synthesized via functionalization with *n*-butylglycidyl ether and subsequently formed into nanoparticles. Chromatographic, spectroscopic, and thermal analytical methods were employed to characterize the novel butylglyceryl pectins (P-OX4) obtained, prior to their formulation into nanoparticles via nanoprecipitation. Nuclear magnetic resonance (NMR) and Fourier transform infrared (FT-IR) spectroscopy confirmed a degree of modification in these materials in the range 10.4–13.6%, and thermal stability studies indicated an increase in both the thermal decomposition onset and glass transition temperature values (compared to those of the original pectin). An increase in the molecular weight and a decrease in the viscosity of P-OX4, when compared to the starting material, were also observed. The resulting nanoformulations were investigated in terms of particle morphology, size and stability, and it was found that particles were roughly spherical, with their size below 300 nm, and a negative zeta potential (−20 to −26 mV, indicating good stability). Having demonstrated the ability to load Doxorubicin at the level of 10%, their potential in drug delivery applications warrants further investigations.

## 1. Introduction

In addition to their low cost, biocompatibility and biodegradability, the ease of chemical modification with various conjugates make polysaccharides very promising materials for the design of various carriers for drug delivery applications [1,2,3]. Among the common types of chemical modifications applied to polysaccharides, the introduction of alkyl groups leads to the adjustment of the hydrophilic lipophilic balance of the macromolecule, which is being used in turn for tuning its interactions with living cells and ultimately for controlling its bioactivity. However, alkylation can also change other important properties of polysaccharides such as their solubility and viscosity in solution [4].

Several reagents can be used for the introduction of alkyl groups to polysaccharides; these include epoxide derivatives such as alkylglycidyl ethers, which react with the free hydroxyl groups, yielding hydrophobically tuned polysaccharides. This modification involves the opening of the epoxide ring in the presence of a strong base as a catalyst (such as potassium *tert*-butoxide, NaOH, or tetrabutylammonium hydroxide) that can facilitate the formation of a strong nucleophile prior to the addition of an alkylating or etherification agent [5].

Pectin, a component of the plant cell wall, is found in the middle lamella and is commonly extracted from the primary cell wall of citrus peel or apple pomace [6,7]. It is a polysaccharide composed primarily of linear polymers of d-galactopyranosyl uronic acid units joined via 1,4 α glycosidic bonds, where the C^6^ carboxylic groups can be esterified to various degrees with methanol. There are also literature reports indicating that this regular structure can be partially acetylated or interrupted with l-rhamnopyranosyl units and with side chains containing other neutral sugars [8]. Pectin has good solubility in water and, due to its excellent biodegradability and biocompatibility, it has been used in targeting various proteins and drugs in various formulations such as hydrogels, films, microspheres, and nanoparticles [9]. Pectin-based systems were mainly used for targeted colonic delivery [10,11,12,13,14], but in some cases also to the brain [15,16]. Previous studies investigating alkylated pectin for drug release applications have considered alkylation with alkyl bromides of varying chain length (butyl, octanoyl, dodecyl, and cetyl) [17].

Rationalized by earlier studies that demonstrated the potential of alkylglycerols in promoting increased drug transport into the brain when co-administered carotidally with model actives [18,19,20], we focused on the preparation and investigation of alkyl glyceryl-modified polysaccharides as drug carriers that could facilitate improved drug access to the brain [21,22]. We report here on the modification of pectin with *n*-butylglycidyl ether and its formulation into nanoparticles via a nanoprecipitation method, with a view to preliminary assess the potential of these materials for future drug delivery applications.

## 2. Materials and Methods 

### 2.1. Materials

Pectin from citrus fruit (MW 17 kDa, 20–34% esterification, cat. no. P9311, batch no. 051M1378V), butylglycidyl ether (BGE) (reagent grade 95%, cat. no. 377031), and NaOH were obtained from Sigma Aldrich (Gillingham, UK). Dimethyl sulfoxide (DMSO), dichloromethane (DCM), trimethylamine, and hydrochloric acid (HCL) was purchased from Fisher Scientific (Loughborough, UK). Doxorubicin hydrochloride (cat. No. AD15377) was obtained from Carbosynth (Compton, UK).

### 2.2. Synthesis of Butylglyceryl-Modified Pectin 

Pectin solution (0.40 g, 2.06 mmol) in deionized water (110 mL) was made alkaline (pH 12) using NaOH (33% *w*/*v*) and stored in a freezer at −18 °C for 7 days. After thawing at room temperature, BGE (5.60 mL, 39.14 mmol) was introduced dropwise into the solution under magnetic stirring; the mixture was stirred further for 16 h at 45 °C, under N_2_ atmosphere. The solution was then neutralized using HCl (10% *v*/*v*) and washed with DCM thrice to remove water-insoluble impurities. Additional purification was done by dialysis (molecular weight cut-off, MWCO 12–14 kDa) against deionized water (10.0 L, exchanged thrice per day) for 72 h, followed by the removal of volatile solvent traces using a Büchi Rotavapor R-200 rotary evaporator (Büchi, Switzerland) and lyophilization (VirTis SP Scientific Sentry 2.0 freeze dryer, Genevac Ltd., Ipswich, UK) with flash-freezing in liquid N_2_. Butylglyceryl pectin (P-OX4) that obtained as a white cotton-like material (yields 85–88%) was then characterized by FT-IR and NMR spectroscopy, gel permeation chromatography (GPC) and thermal analysis.

### 2.3. Characterization of Butylglyceryl-Modified Pectin

The resulting lyophilized products were characterized by NMR spectroscopy using a JEOL Eclipse 400+ instrument (JEOL, Welwyn Garden City, UK; 400 MHz for ^1^H- and 100 MHz for ^13^C-NMR). The samples were dissolved in D_2_O, with TMS (0.2% *v*/*v*) as reference. DS was calculated from the ^1^H- NMR spectra of P-OX4. FT-IR spectra were recorded using a Nexus Euro infrared spectrometer (Thermo Fisher Scientific, Hemel Hempstead, UK) equipped with a diamond crystal ATR Smart Orbit accessory. Thermogravimetric analysis (TGA) and differential scanning calorimetry (DSC) were performed using TG 209 F1 Libra (NETZSCH, Germany) and DSC 214 Polyma (NETZSCH, Selb, Germany) instruments, respectively.

The viscosity of both pectin and P-OX4 was tested using a Gilmont Falling Ball Viscometer (GV-2100, Gilmont Instruments Inc., Barrington, IL, USA). Samples were dissolved in deionized water at 1% concentration. The viscometer constant was 0.3, the density of the glass ball 2.53 g/mL and the density of the liquid 1.0 g/mL. The polymer solution was poured into the tube until nearly full (5 mL) and the ball was carefully added and allowed to drop. The time taken for the ball to travel through the sample solution was recorded.

Gel permeation chromatography (GPC) was performed using a Waters Alliance GPC 2000 system (Waters Corporation, Milford, MA, USA) equipped with a PL-aquagel-OH column (8-μm particle size; 40 Å pore type) and refractive index detector, under controlled temperature conditions (30 °C). Either a mixture of water/methanol (8:2, *v*/*v*) or 100% water (high-performance liquid chromatography, HPLC grade) was used as the eluent at a flow rate of 0.5 mL/min. The molecular weight (MW) was estimated based on the calibration conducted using pullulan standards (Showa Denko, New York, NY, USA) of MWs 0.6 × 10^4^, 1 × 10^4^, 2.17 × 10^4^, 4.88 × 10^4^, 11.3 × 10^4^, 21 × 10^4^, 36.6 × 10^4^, and 80.5 × 10^4^ g/mol. Statistical analysis was performed using the IBM SPSS Statistics Version 22 software (SPSS Inc., Chicago, IL, USA, 2013). The results are expressed as mean ± standard deviation (SD); to determine statistical significance, the p values were set at 0.05 unless stated otherwise.

### 2.4. Formulation of Nanoparticles from Butylglyceryl-Modified Pectin

Butylglyceryl-modified pectin (P-OX4) was dissolved in DMSO (2 mL) at different concentrations (2, 5, 10, and 15 mg/mL); the solutions were filtered through a 0.2-μm nylon membrane Whatman syringe filter and then introduced from a syringe to ultrapure water (8 mL) as the dispersion phase, with vigorous magnetic stirring. The resulting nanoparticles were then either centrifuged (Beckman Coulter, Fullerton, CA, USA, 70.1 Ti rotor; 40,000 rpm; 164,391 *g*; 30 min) or dialyzed (MWCO 12 kDa) against deionized water (10 L; three exchanges per day) for 72 h. After purification, the products were lyophilized. The nanoparticles were obtained (yield 42–81%) and redispersed (0.5 mg/mL) in either ultrapure water or PBS (0.9% NaCl; pH 7.4) for characterization of the nanoformulation by using dynamic light scattering (DLS), and electrophoretic mobility (EPM).

### 2.5. Physical Characterization of Butylglyceryl Pectin Nanoparticles

The hydrodynamic diameter of nanoparticles was measured by DLS using a Malvern ZetasizerNano ZS instrument (Malvern Instruments Ltd., Worcestershire, UK) equipped with a 633-nm He-Ne laser and controlled by Zetasizer v7.01 software (Malvern Instruments Ltd., Worcestershire, UK, 2011); the measurements were conducted at a scattering angle of 173° (Malvern Instruments, Malvern, UK). The samples were analyzed in triplicate at 25 °C and the data was expressed as the Z-average mean (Z-av.) and polydispersity index (PDI). Electrophoretic mobility (EPM) measurements were conducted using the same instrument to determine the zeta potential (ZP) of nanoparticles; samples were measured in folded capillary cells (DTS1070, Malvern) and data was processed according to Smoluchowski’s model (Henry’s function *f*(ka) = 1.5).

Scanning electron microscopy (SEM) was employed to investigate the morphology of the nanoparticles. The samples were prepared by redispersing the dried nanoparticles in ultrapure water (10 mg/mL). A droplet was deposited onto a metallic stub and dried prior to coating with gold alloy in an argon atmosphere by using a Q150RES sputter coater equipment (Quorum Technologies Ltd., Ashford, UK).

### 2.6. Loading Capacity of Butylglyceryl Pectin Nanoparticles

The loading capacity of the nanoparticles for model actives such as Doxorubicin was investigated following loading into nanoparticles via nanoprecipitation. Doxorubicin was purchased as hydrochloride salt and the removal of hydrochloride group was performed by treatment with triethylamine as described: Triethylamine (0.1 mL) was introduced to an aqueous solution of Doxorubicin HCl (10 mL; 50 mg/mL). The mixture was stirred for 1 h and washed with chloroform thrice (approx. 150 mL). The combined organic layers were dried by stirring overnight with anhydrous magnesium sulphate to remove water trace, and the remaining organic solvent was removed by a Büchi Rotavapor R-200 rotary evaporator (Büchi, Switzerland), affording the free base of Doxorubicin as red powder (yield 71–80%). 

Doxorubicin solution (0.5 mL; 0.4 mg/mL) in DMSO was mixed with polymer solution during nanoparticles preparation. The resulting nanoparticles were separated by ultracentrifugation (40,000 rpm; 164,391 g; 30 min, 20 °C, Beckman, rotor 70.1 Ti); the pellets were lyophilized and weighted; the supernatant was measured to determine the amount of unbound model actives (which was used to calculate the amount of bound model actives) by UV/Vis (measuring at 486 nm for Doxorubicin) and the results were plotted against the calibration curves. The drug loading was calculated using Equation (1) [23,24]:

(1) $$DL\;\left(\%\right)\;=\frac{weight\;of\;drug}{weight\;of\;nanoparticles\;}\;\times \;100$$

## 3. Results and Discussion

**Figure 1:**
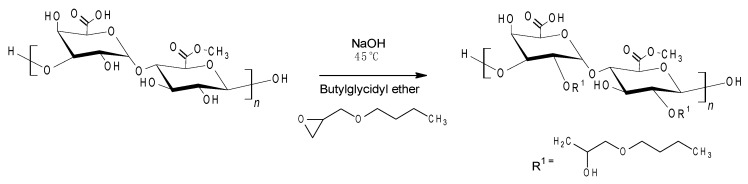
Synthesis of butylglyceryl pectin (P-OX4).

A range of butylglyceryl-modified pectins (P-OX4) were synthesized by reacting commercially available pectin (low degree of esterification) with BGE (Figure 1), via a nucleophilic substitution reaction in a strong alkaline environment, following a method previously described in the literature for chitosan. The strong alkaline environment was necessary to convert the hydroxyl groups of pectin into alcoholates that react further with the oxirane ring via a nucleophilic substitution reaction [21,25]. Slow freezing in alkaline prior to alkylation was previously reported to facilitate the chemical modification of polysaccharides by enhancing their solubility as a result of breaking the intra and intermolecular hydrogen bonds, disturbing the ordered molecular structure and decreasing crystallinity [26,27]. P-OX4 were obtained as white, low-density, cotton-like solids (yield 85–88%). 

**Figure 2:**
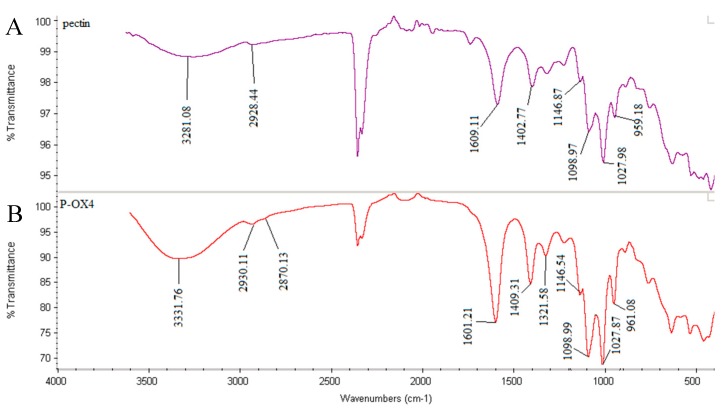
Fourier transform infrared (FT-IR) spectra of: (**A**) pectin, and (**B**) P-OX4.

The alkylation of pectin was confirmed by FT-IR and NMR spectroscopic analyses. The broad and intense absorption band at 3331–3281 cm^−1^ (appearing in the spectra of both pectin and P-OX4, Figure 2) can be assigned to O–H stretching absorption due to the inter and intramolecular hydrogen bonding present in the galacturonic acid units [28,29]. The bands at 2930–2928 cm^−1^ can be attributed to C–H vibrations including CH, CH_2_, and CH_3_ stretching. The low-esterified pectin used in this study did not exhibit a distinct O–CH_3_ stretching band, normally appearing between 2950 and 2750 cm^−1^ and attributed to the methyl esters of galacturonic acid, as reported in the literature [30,31], most likely masked by the large O–H stretching bands appearing in the region (3600–2500 cm^−1^). Two bands attributed to the carboxylate groups (COO^−^) were observed in both pectin and P-OX4: an asymmetric stretching band near 1609–1601 cm^−1^, and a weaker symmetric stretching band near 1409–1402 cm^−1^ [30,32]. Comparing the FT-IR spectrum of P-OX4 with that of pectin, a higher intensity of the bands at 1027 and 1098 cm^−1^ (attributed to the formation of additional ether linkages C–O–C) can be observed for P-OX4. The band between 2400 and 2300 cm^−1^ (appearing in both samples, with variable intensity) was due to the atmospheric CO_2_ [33].

**Figure 3:**
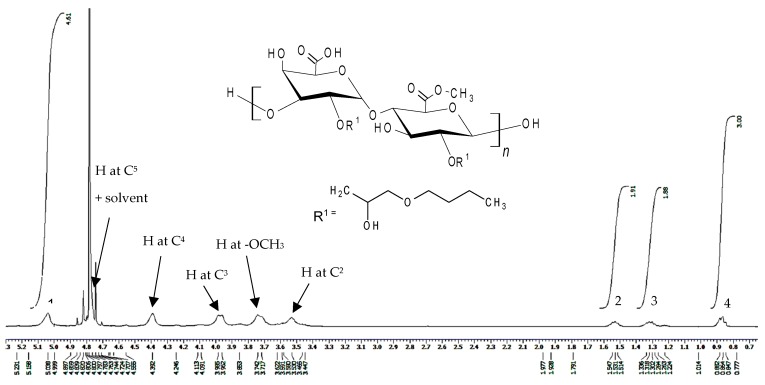
Proton nuclear magnetic resonance (^1^H-NMR) spectrum of P-OX4 in D_2_O (5 mg/mL).

The successful alkylation of pectin was also confirmed by NMR spectroscopy. The ^1^H-NMR spectrum of P-OX4 (Figure 3) shows the presence of peaks characteristic of the sugar repeating unit (δ 5.0 ppm for the anomeric proton, δ 3.7 ppm for the methoxy protons of esterified pectin, and δ 3.5–4.8 for the backbone protons) [32] and alkyl groups of the butylglyceryl-pendant chain (δ 0.9, 1.3, and 1.5 ppm). ^1^H-NMR spectra of n-butylglycidyl ether (BGE) was also studied (Figure S1, Supplementary Materials) to facilitate the confirmation. 

The ^1^H-NMR spectrum was also employed to calculate the degree of substitution (DS, defined as the number of butylglyceryl chains attached to 100 glucopyranose residues of pectin; e.g., 100% indicates that one OH group was substituted in each sugar residue, with a maximum value of 200% when all available OH groups in each sugar residue of pectin were fully substituted), using Equation (2):

(2) $$DS\;\left(\%\right)=\;\frac{A\;\times \;100}{3B}$$

where

*DS* (%)   Degree of substitution (number of butylglyceryl chains attached to 100 sugar residue of pectin)
*A*     Integral value of the signal assigned to the alkyl chain-end CH_3_ group (δ 0.9 ppm)
*B*     Integral value of the signal assigned to the anomeric C^1^ proton of the glucopyranose ring (δ 5.0 ppm)

The formula used to calculate DS for P-OX4 was derived using the peak integrals attributed to the end CH_3_ group alkyl chain (δ 0.9 ppm) and the anomeric proton at C^1^ (δ 5.0 ppm). The results indicate degrees of substitution around 10.4–13.6%, a significant increase compared to previous literature data reporting very low values obtained for alternative alkylation reactions (e.g., Morris et al. alkylated pectin with *p*-carboxybenzyl bromide in an aqueous alkali medium, with very low DS values, <0.1% [34]; Liang et al. reported alkylation with alkylglycerols of various alkyl chain lengths—hexyl, dodecyl, or octadecyl—in the presence of TBAOH, with DS values that ranged from 0.6 to 4 [19]). The reaction scheme (Figure 3) indicates that the substitution mainly takes place at C^2^, which is the most reactive OH group [35,36,37].

**Figure 4:**
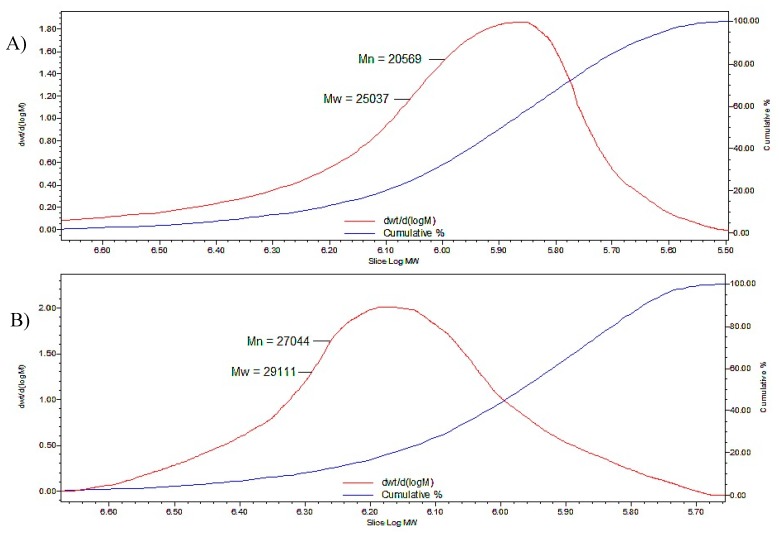
GPC data (molecular weight distribution and cumulative percent curves) for: (**A**) pectin and (**B**) P-OX4.

GPC was performed to investigate the MW of P-OX4 and the results showed an increase of the MW following modification (Table 1), which confirms the attachment of butylglyceryl-pendant chains to the polysaccharide backbone of pectin (comparable to the results discussed by Devedec et al. [38]). The polymer polydispersity index (PDI), as obtained by GPC and reported here, is based on the ratio between MW and molecular number (Mn) and indicates the MW distribution of a polymer, with values close to 1 indicating a narrow polymer distribution (i.e., more monodispersed). GPC chromatograms of P-OX4 vs. pectin are exemplified in Figure 4.

**Figure 5:**
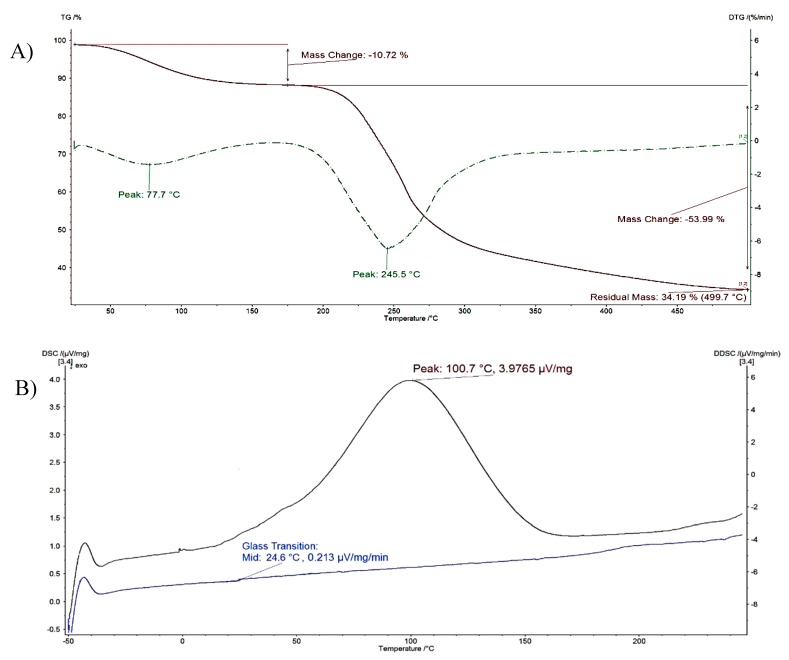
Typical thermal analysis results for P-OX4: (**A**) Thermogravimetric analysis (TGA) thermogram (first derivative thermogravimetry (DTG) represented as dotted line); and (**B**) Differential scanning calorimetry (DSC) curves: First run—top, and second run—bottom).

An example of the typical results obtained from thermal analysis is presented in Figure 5. Two main steps corresponding to different mass-loss processes were identified in the TGA thermograms of P-OX4 (% mass loss represented as a function of temperature). The first step was caused by the evaporation of the humidity still present in the freeze-dried product (~11% water content), whereas the second step (derivative thermogravimetry, DTG peak at ~245 °C, with a mass loss >53%) was attributed to the thermal decomposition of the material, in accordance to literature [39]. 

Table 2 summarizes the data obtained for all samples and includes the starting material (pectin). The thermal analysis data confirms the expected increase in hydrophobicity following the modification with BGE; the measured water content is inversely proportional to the DS (pectin containing more water, ~16%, compared to P-OX4 with <12%). The DTG peak corresponding to water evaporation in P-OX4 derivatives appeared at a higher temperature.

The glass transition temperature (*T*_g_) of pectin was also noted to shift to higher temperatures following the substitution with BGE (Figure 5). This can perhaps be associated with an increase in the bulkiness of the molecular structure following the insertion of the pendant chains, ultimately leading to a decrease in mobility. Moisture has also been reported to act as a plasticizer, leading to an increase in free volume and weakening the inter-chain interactions, which in turn lead to a decrease of the *T*_g_ of amorphous solids such as the pectin [40,41,42]. The *T*_g_ values of pectin reported in the literature vary considerably. Basu et al. investigated the *T*_g_ of pectin relative to its moisture content, and found that the value decreased from 16.8 to −24.6 °C when the moisture content increased from 8 to 20% [40]. Mishra et al. studied the effect of graft polymerization of pectin with polyacrylamide, and discovered that the *T*_g_ of pure pectin decreased after the grafting from 95 to 53 °C [43]. As *T*_g_ values depend on the composition of each particular sample, the differences observed could potentially be explained variations in the batch composition, MW and water content [44,45].

A falling-ball viscometer was used to assess the alkylation effect on the viscosity of pectins, which was calculated using Equation (3):

η = *K*(ρ*_t_* − ρ)·*t* (3)

where

η      Viscosity (in centipoise, cp)
ρ*_t_*      Density of ball (g/mL)
ρ      Density of liquid (g/mL)
*t*      Time of the ball descent in the liquid (mins)
*K*      Viscometer constant (depending on ball size)

The results show that P-OX4 has a reduced viscosity compared to that of pectin (Table 3), possibly due to reduced inter and intramolecular hydrogen bonding as a result of the introduction of the bulky alkyl glyceryl chains [46].

**Figure 6:**
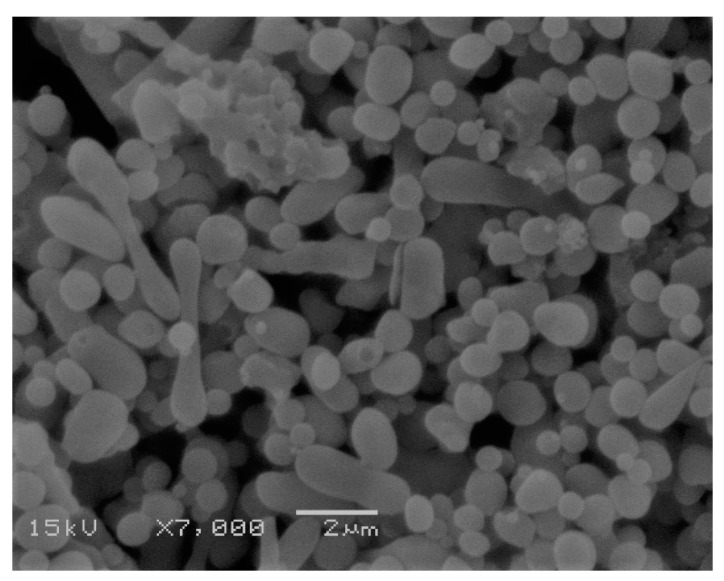
Scanning electron microscopy (SEM) micrograph of lyophilized P-OX4 particles (scale bar = 2 µm).

P-OX4 was further investigated for its ability to form nanoparticulates that might be suitable as drug vehicles, and we found that nanoprecipitation (previously applied for dextrans [47,48]) is also one of the most appropriate methods applicable to hydrophobized pectins. P-OX4 dissolved in DMSO is simply subjected to dialysis under controlled conditions, when the material easily self-assemble into micro and nanoparticulates having a close-to-spherical morphology (Figure 6). For comparison purposes, we attempted to prepare pectin nanoparticles using the same technique employed for P-OX4 (i.e., nanoprecipitation), but we were not successful as the solubility of normal pectin at similar concentrations is very different from P-OX4.

The process has been repeated at different polymer concentrations, and the results indicated that P-OX4 (5 mg/mL) exhibited the smallest size (though the difference was not significant; ANOVA, *p* < 0.05) and had good polydispersity index (indicative of uniform distribution). Particles around 250 nm showing good polydispersity (PDI < 0.5) and negative zeta potential (−20 to −26 mV, indicative of stable formulations [49]) can be prepared without difficulty (Table 4). It has been observed that an increase in concentration above 10 mg/mL leads to an increase in size.

In a preliminary study, a formulation of nanoparticles that exhibited the smallest size (conc. of 5 mg/mL P-OX4) was selected, and doxorubicin was employed as a model active in order to investigate the drug loading ability of these nanoparticles. The chemotherapeutic agent was loaded during the nanoprecipitation stage, and the degree of loading was determined by UV–Vis to be 10.4 ± 1.8% (*n* = 3; ±SD), similar to previous reports on doxorubicin loaded into pectin nanoparticles via microemulsification with 18% drug loading) [50]. The size of the loaded nanoparticles was investigated using DLS, and the results indicated a significant increase in size (ANOVA, *p* < 0.05), to about 280.1 ± 4.2 nm, with −19.8 ± 2.1 mV zeta potential, and having 0.10 ± 0.05 polydispersity index (*n* = 3; ±SD).

## 4. Conclusions

Butylglyceryl-modified pectins (P-OX4) with increased amphiphilicity can be easily prepared via a typical nucleophilic substitution reaction with *n*-butylglycidyl ether; the successful modification, with a degree of substitution of around 10.4–13.6%, was confirmed by FT-IR and NMR spectroscopy as well as by GPC. Results of the thermal stability studies (TGA, DSC) indicated that the modification led to an increase in both the glass transition (*T*_g_) and decomposition onset temperature values compared to those of pectin, most likely as a result of the macromolecular motion-limiting effect of the newly introduced pendant chains. Our results demonstrate that these novel materials can be easily formulated—by nanoprecipitation, via self-assembly—into stable formulations containing particulates below 300 nm size, with good polydispersity index and a drug loading degree of 10.4 ± 1.8% (when loaded with Doxorubicin). We believe these characteristics warrant further investigations into the potential of butylglyceryl pectins (P-OX4) in drug delivery applications.

## Figures and Tables

**Table 1:** Average molecular weight data for P-OX4 derivatives, as determined by gel permeation chromatography (GPC) (*n* = 3, ±SD).

Compound	Number Average Molecular Weight (Mn)	Weight Average Molecular Weight (Mw)	Polydispersity Index (PDI)
Pectin	20,569 ± 276	25,037 ± 128	1.22 ± 0.10
P-OX4	27,044 ± 298	29,111 ± 252	1.13 ± 0.06

**Table 2:** Thermogravimetric analysis (TGA) results obtained for P-OX4 derivatives (*n* = 3, ± SD).

Material	Water Evaporation		Decomposition		Glass Transition, *T*_g_ (°C)
	DTG Peak (°C)	Mass Loss (%)	DTG Peak (°C)	Mass Loss (%)	
Pectin	56.2 ± 6.9	16.32 ± 8.1	215.4 ± 6.1	17.62 ± 7.4	22.4 ± 1.5
P-OX4	77.7 ± 7.4	10.72 ± 8.2	245.5 ± 8.9	53.99 ± 7.1	24.7 ± 1.6

**Table 3:** Results of viscosity measurements (*n* = 3; ±SD).

Samples	Time of Descent *t* (min)	Viscosity η (cp)
Pectin	0.80 ± 0.1	0.37 ± 0.1
P-OX4	0.61 ± 0.1	0.28 ± 0.1

**Table 4:** Characteristics of nanoparticles prepared from P-OX4 by nanoprecipitation (*n* = 3; ±SD).

Polymer	Concentration (mg/mL)	Diameter (nm)	Polydispersity Index	Zeta Potential (mV)
P-OX4	2	253.9 ± 11.1	0.18 ± 0.09	−20.4 ± 2.1
	5	251.1 ± 13.9	0.10 ± 0.08	−22.7 ± 3.0
	10	252.9 ± 12.4	0.20 ± 0.10	−24.2 ± 2.8
	15	279.9 ± 18.9	0.21 ± 0.12	−26.2 ± 3.3

## Supplementary Materials

The following are available online at https://www.mdpi.com/2073-4360/11/5/789/s1, Figure S1: ^1^H-NMR spectra of n-butylglycidyl ether (BGE) in deuterated methanol (CD_3_OD) (10 mg/mL).
