# Supplementary figures and images for: Butylglyceryl Pectin Nanoparticles: Synthesis, Formulation and Characterization
Source: Polymers (Basel). 2019 May 2;11(5):789. doi: 10.3390/polym11050789 (PMC6571649; doi:10.3390/polym11050789)

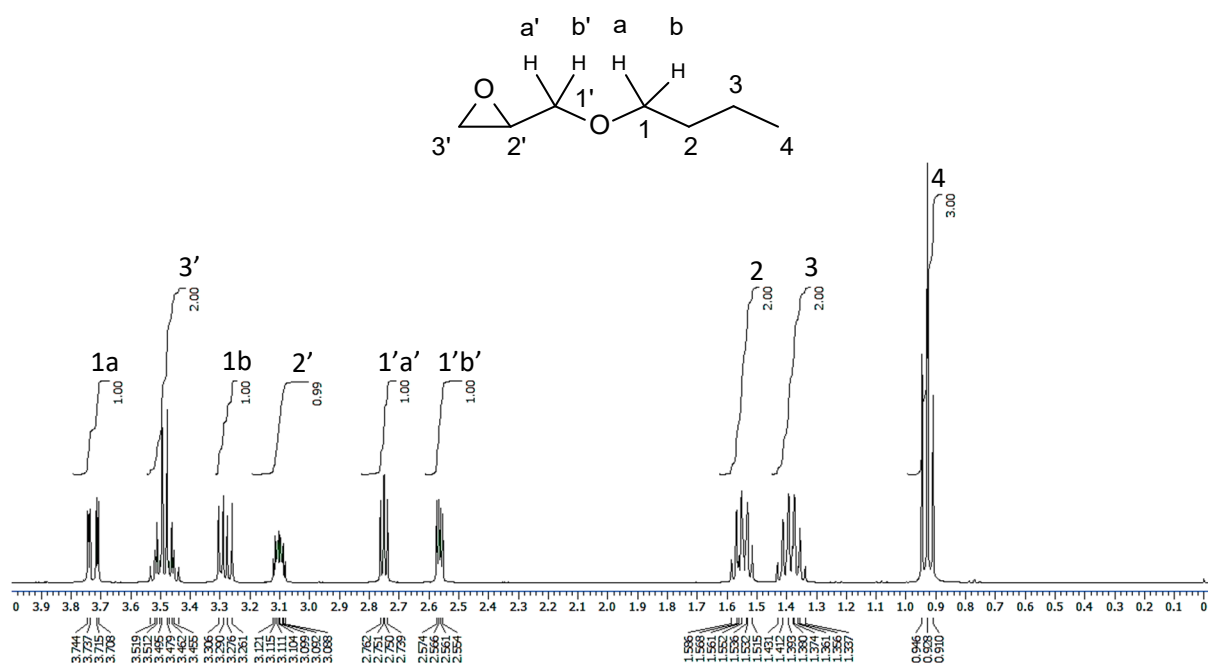

**Figure S1.**  $^1\text{H-NMR}$  spectra of n-butylglycidyl ether (BGE) in  $\text{CD}_3\text{OD}$  (10 mg/mL).

Supplement: Supplementary file 1 [file polymers-11-00789-s001.pdf]
